# Contextual factors of implementing *APOL1* genetic testing into living kidney donor clinical evaluation

**DOI:** 10.1007/s00103-025-04068-8

**Published:** 2025-06-11

**Authors:** James L. Merle, Marissa C. Kuo, Jessica Gacki-Smith, Akansha Agrawal, John Friedewald, Elisa J. Gordon, Justin D. Smith

**Affiliations:** 1 Department of Population Health Sciences, University of Utah School of Medicine, Salt Lake City, UT USA; 2 Department of Surgery, Vanderbilt University Medical Center, Nashville, TN USA; 3 Northwestern University Feinberg School of Medicine, Chicago, IL USA; 4 Departments of Surgery and Medicine, Northwestern University Feinberg School of Medicine, Chicago, IL USA; 5 Department of Surgery, and Center for Biomedical Ethics and Society, Vanderbilt University Medical Center, 1161 21st Avenue South, CCC-4309 MCN, 37232-2730 Nashville, TN USA

**Keywords:** Black, Counseling, Implementation science, Informed consent, Culturally targeted intervention, Dunkelhäutige Lebendnierenspender, Beratung, Implementierungswissenschaft, Informierte Zustimmung, Kulturell ausgerichtete Intervention

## Abstract

**Background:**

Living donor kidney transplantation (LDKT) is the preferred treatment for patients with end-stage kidney disease, offering longer graft survival and improved quality of life. However, LDKT poses risks to living donors. Black living donors face disproportionately higher risks of postdonation kidney disease than White counterparts, necessitating deeper understanding of the factors contributing to this disparity. This study evaluated the implementation of the *APOL1* Genetic Testing and Counseling Program at two transplant centers to improve donors’ informed decision-making, which entailed examining the contextual factors influencing its adoption and sustainment.

**Methods:**

We conducted a mixed-methods evaluation involving semistructured interviews guided by the Consolidated Framework for Implementation Research and surveys with transplant nephrologists to identify facilitators and barriers of intervention implementation.

**Results:**

Eleven nephrologists participated. Key facilitators included alignment with clinical priorities, strong organizational support, and value attributed to the intended goal of enhancing donors’ informed consent process and to the integration of culturally sensitive counseling practices. Key barriers included time constraints and the need for clear evidence-based guidelines. Participants reported high acceptability, appropriateness, feasibility, and sustainability of the *APOL1 *Program.

**Conclusion:**

Our study identified facilitators and barriers that should be addressed to ensure the *APOL1* program’s sustainment and potential to improve donors’ informed consent. Future research should leverage system-level implementation strategies to overcome identified barriers when taking the *APOL1* Genetic Testing and Counseling Program to scale.

**Supplementary Information:**

The online version of this article (10.1007/s00103-025-04068-8) contains supplementary material, which is available to authorized users.

## Abstract (de)

**Hintergrund:**

Die Nierentransplantation durch einen Lebendspender („living donor kidney transplantation“, LDKT) ist die bevorzugte Behandlung für Patienten mit Nierenerkrankungen im Endstadium, da sie eine längere Transplantatüberlebensdauer und eine verbesserte Lebensqualität bietet. Die LDKT birgt jedoch Risiken für die Lebendspender. Dunkelhäutige Lebendspender sind einem unverhältnismäßig höheren Risiko für Nierenerkrankungen nach der Spende ausgesetzt als hellhäutige Spender, was ein tieferes Verständnis der Faktoren erfordert, die zu dieser Ungleichheit beitragen. In dieser Studie wurde die Umsetzung des Gentest- und Beratungsprogramms *APOL1* in zwei Transplantationszentren evaluiert, um die informierte Entscheidungsfindung der Spender zu verbessern. Dabei wurden die Kontextfaktoren untersucht, die die Akzeptanz und Nachhaltigkeit des Programms beeinflussen.

**Methoden:**

Wir führten eine Mixed-Method-Evaluierung durch, die halbstrukturierte Interviews, die sich am „Consolidated Framework for Implementation Research“ orientierten, und Befragungen von Transplantationsnephrologen umfasste. Die Studie ermittelte die Faktoren, die die Umsetzung der Intervention erleichtern bzw. behindern.

**Ergebnisse:**

Elf Nephrologen nahmen an der Studie teil. Zu den wichtigsten erleichternden Faktoren gehörten die Ausrichtung an klinischen Prioritäten, eine starke organisatorische Unterstützung und der Wille, den Prozess der informierten Einverständniserklärung zu verbessern und kultursensible Beratungspraktiken zu integrieren. Zu den wichtigsten Hindernissen gehörten Zeitbeschränkungen und der Bedarf an klaren evidenzbasierten Richtlinien. Die Teilnehmenden berichteten von einer hohen Akzeptanz, Angemessenheit, Durchführbarkeit und Nachhaltigkeit des *APOL1*-Programms.

**Schlussfolgerung:**

Unsere Studie ermittelte Faktoren, die die Umsetzung des Programms erleichtern und erschweren, und die angegangen werden sollten, um die Nachhaltigkeit des Programms *APOL1* und sein Potenzial zur Verbesserung der informierten Einverständniserklärung von Spendern zu gewährleisten. Zukünftige Forschung sollte Umsetzungsstrategien auf Systemebene nutzen, um die identifizierten Hindernisse zu überwinden, wenn das Gentest- und Beratungsprogramm *APOL1* in großem Maßstab umgesetzt wird.

## Background

Living donor kidney transplantation (LDKT) is the optimal treatment for patients with end-stage kidney disease. LDKT provides recipients with longer graft and patient survival, as well as improved quality of life when compared to deceased donor kidney transplantation and dialysis [1, 2]. Although all living kidney donors (LDs) have a higher risk of kidney disease post-donation compared to healthy nondonors, Black LDs have a disproportionately higher risk of kidney disease post-donation than do White LDs [3–5]. Little is known about factors contributing to this disparity. Evidence suggests that genetic markers may contribute to these post-donation disparities in chronic kidney disease (CKD) [3, 4]. Transplant teams are committed to protecting LDs’ safety because LDs undergo harm for no medical benefit to themselves [6, 7]. Ensuring that LDs comprehend their risks of donation is essential for protecting LDs’ autonomy through informed decision-making [5–7].

*Apolipoprotein* L1 (*APOL1*) gene risk variants, which are permanent changes to the DNA, are associated with increased risk of developing nondiabetic CKD in the general population [8]. These risk variants evolved nearly 4000 years ago within sub-Saharan Africa as a protection against sleeping sickness. Thus, individuals of African ancestry are most likely to carry *APOL1* risk variants [8]. In the United States, 13% of Black individuals have two *APOL1* risk variants, which confers a 15% lifetime risk of developing CKD [8, 9]. The relationship between *APOL1* risk variants and CKD may help to explain disparities in post-donation CKD among LDs of African ancestry.

Accordingly, some transplant programs use *APOL1 *genetic testing as part of the LD clinical evaluation process to ascertain LD safety [10, 11]. Because the relationship between *APOL1* and post-donation kidney disease in LDs is currently associational, many in the transplant field are awaiting population-level data from the ongoing *APOL1* Long-Term Kidney Transplantation Outcomes Network (APOLLO) study on whether *APOL1* high-risk genotypes are associated with poorer kidney outcomes in LDs after nephrectomy [12]. Without clinical guidelines, transplant providers vary in their use and understanding of the clinical indications for *APOL1* testing [13].

To address these problems, we developed the *APOL1 Genetic Testing and Counseling Program* (henceforth called the *APOL1 *Program), which was implemented in two large-volume transplant centers as a clinical trial (ClinicalTrials.gov Identifier: NCT04910867; registered May 8, 2021). *APOL1* Program components include screening of all potential LDs for African ancestry via three self-report questions; a culturally adapted conversational chatbot, Gia® (Genetic Information Assistant; Labcorp Genetics Inc., Durham, NC, USA), about *APOL1* to provide potential LDs of African ancestry with foundational knowledge immediately prior to their nephrologist clinic visit [14]; nephrologist training in *APOL1* genetic testing and counseling (the *APOL1* Counseling Training Program); nephrologist encouragement of all LDs of African ancestry to undergo *APOL1* testing; *APOL1* testing of all potential LDs of African ancestry who consent; and an *APOL1 *educational brochure that is provided to LDs. The study protocol details the study intervention components and implementation strategies [15]. The *APOL1* Program was intended to serve as a model for implementing genetic testing and genetic counseling in a culturally sensitive manner.

Upon deploying interventions, organizations commonly encounter contextual factors (i.e., barriers or facilitators) that impede or enable implementation [16, 17]. Research evaluating the implementation of genetic testing interventions has focused on assessing genetic testing in the setting of hereditary conditions, pharmacogenetic testing and genomics, and screening programs [18–20]. Commonly identified barriers to implementing genetic testing programs include: cost of testing, lack of provider knowledge about the test, and the need for educational materials for providers about the clinical utility of test results [19, 21]. Among nephrologists, documented barriers to genetic testing include lack of education around interpreting and using genetic tests in counseling [22–24]. Commonly identified facilitators include: availability of evidence supporting the clinical utility of the test, prior exposure to similar genetic testing programs, and having a trusted clinician provide counseling about the test [14, 16, 18–20, 25]. Among nephrologists, studies suggest support for implementing genomic testing in a multidisciplinary fashion [22–24].

Few studies have evaluated barriers to genetic testing for organ transplantation. In one study evaluating perspectives on the utility of pharmacogenetic testing post-transplant, barriers included lack of provider knowledge, lack of evidence demonstrating clinical utility, and high patient volume [20]. No research has examined facilitators to genetic testing in transplantation. Our focus group study of prior LDs of African ancestry found support for undergoing *APOL1* testing and using chatbots to facilitate learning about *APOL1* prior to genetic testing [26].

Evaluating the implementation of interventions is valuable for identifying intervention-unique barriers or facilitators [27, 28]. Evaluating factors affecting intervention sustainment (i.e., the continued delivery over time) is crucial to increasing the likelihood of the intervention’s long-term delivery [29]. To date, no studies have evaluated transplant provider–perceived barriers and facilitators to implementing *APOL1* genetic testing and counseling into LD clinical evaluation. This study assessed the contextual factors affecting implementation of the *APOL1* Program.

### Theoretical framework

The Consolidated Framework for Implementation Research (CFIR) is an evidence-based determinant framework used to assess intervention implementation in diverse contexts, including health care, community-based settings, and education [16, 30, 31]. We chose the updated CFIR 2.0 to guide our evaluation because it has synthesized evidence from many disciplines into one framework. The CFIR comprises 39 constructs organized into five domains: innovation (i.e., factors affecting the intervention being implemented), inner setting (i.e., internal organizational influences on implementation), outer setting (i.e., influences on implementation external to the institution), individuals (i.e., roles and characteristics of individuals involved in intervention deployment), and process (i.e., activities and strategies involved in the implementation process) [31].

## Methods

This study was conducted within a nonrandomized clinical trial, including a pre-/post-implementation evaluation. Conducted as a type 2 hybrid effectiveness–implementation study, the trial simultaneously evaluated the effectiveness of the *APOL1* Program and the prospective implementation strategy [20]. This paper presents one of several implementation evaluation analyses of the *APOL1* Program, focusing on transplant nephrologists’ perceptions of implementing the *APOL1 *Program. This mixed-methods analysis assessed nephrologists’ perceived barriers and facilitators, acceptability, appropriateness, feasibility, and sustainability (i.e., likelihood of continuous use of the program, as intended, over time) of the *APOL1* Program.

### Study design

This prospective longitudinal study entailed data collection through a mixed-methods, concurrent design. Qualitative and quantitative data were collected through semistructured interviews and surveys, respectively. We used the Standards for Reporting Qualitative Research for quality reporting of qualitative research [32].

### Setting and participants

The *APOL1* Program is being conducted at transplant centers at Northwestern University (NU; Chicago, IL) and Georgetown University (GU; Washington, DC) in 2021–2026. This study was approved by the NU Institutional Review Board of record (STU00214038) and the GU Institutional Review Board (STUDY00003752) in accordance with the Declaration of Helsinki of 1975. The *APOL1* Program was implemented at NU on August 18, 2022, and at GU on August 19, 2022. Eligible participants included all transplant nephrologists, recruited through purposive sampling.

### Study procedures and data collection

We conducted telephone/Zoom semistructured interviews to assess transplant nephrologists’ perceived contextual factors (barriers and facilitators) affecting the integration and use of the *APOL1* Program in LD clinical evaluation. Interviews occurred at multiple time points: within 1–2 months of initiating *APOL1* Program implementation, a year thereafter, and 2 years thereafter [30]. Initial interviews were completed between August 30, 2022, and October 28, 2022. Interviews lasted approximately 30 min (range: 16–45 min) and were audio-recorded. Validated quantitative surveys were administered at all time points, immediately following the semistructured interviews, to assess acceptability, appropriateness, and feasibility [33]. A survey on sustainability [34] was administered at the end of each participant’s last interview.

### Measures

The qualitative interview guide was informed by the CFIR and included a subset of CFIR constructs most relevant to the *APOL1* Program. The Supplemental File presents our interview guide. The acceptability of intervention measure (AIM) assessed intervention acceptability [33]. The intervention appropriateness measure (IAM) assessed intervention appropriateness [33]. The feasibility of intervention measure (FIM) assessed intervention feasibility [33]. Across these measures, our sample had good internal consistency (α = 0.75, 0.85, and 0.94, respectively). The Clinical Sustainability Assessment Tool, short form (CSAT-Short) assessed intervention sustainability [33–35]. At the end of the CSAT survey, nephrologists were asked, “Do you want to continue delivering the *APOL1* genetic testing program?” (response options: yes/no). Our sample had good internal consistency overall (α = 0.97) and for each subscale (range: α = 0.90–α = 0.95).

### Data analysis

#### Qualitative analysis.

All interviews were transcribed verbatim. Transcripts were analyzed and coded using framework analysis to categorize the contextual factors within CFIR [36, 37]. Each contextual factor was coded as a barrier, facilitator, both (could either facilitate or hinder), or unsure/unspecified. Each factor was also coded regarding whether it was related to *APOL1* Program sustainability after the trial ended and whether it was related to ethical and/or equity considerations, as identified elsewhere [16].

An initial codebook was developed based on selected constructs within CFIR 2.0 and was iteratively refined through discussion with the research team [31]. Inductive coding was used to capture emergent factors beyond CFIR codes (i.e., codes related to structural/systemic oppression and equity). Coding training was provided by the senior authors (EJG and JDS) [38]. Each transcript was double-coded, and consensus meetings were held by the co-first authors (JLM and MCK). Discrepancies in coding were resolved through discussion, and unresolved discrepancies were adjudicated by the senior authors. Coding was conducted in MAXQDA24, a qualitative data analysis software program [39].

Results were analyzed in MAXQDA using a matrix of CFIR codes whereby rows represent CFIR constructs and columns represent individual participants. We also performed trajectory analysis via the coding matrix to compare whether and how the data, grouped by CFIR domain and by site, changed over time [40]. Further, we compared contextual factors between sites (i.e., NU versus GU) and differences within interviewee transcripts across time.

#### Quantitative analysis.

Descriptive statistics were used to analyze the survey data, including frequencies, means, standard deviations, and ranges. Analyses were conducted in RStudio*.*

## Results

### Participant characteristics

Eleven transplant nephrologists participated. Most were female (7/11, 64%), and most had been in clinical practice for more than 10 years (8/11, 73%; Table 1).

**Table 1:** Participants’ demographic and clinical characteristics, *N* = 11

Variable	*n* (%)
*Age, years*	Mean [SD] (range)
	44.1 [4.4] 38–51
*Sex*	
Female	7 (64)
Male	4 (36)
*Ethnicity*	
Not Hispanic or Latino	9 (82)
Hispanic or Latino	2 (18)
*Race*	
Asian	5 (45)
White	4 (36)
Black or African American	1 (9)
Multiracial (White and Black/African American)	1 (9)
*Years in practice*	
1–5 years	1 (9)
6–10 years	2 (18)
11–20 years	7 (64)
21–25 years	1 (9)
*Study site*	
Georgetown University	6 (55)
Northwestern University	5 (45)

### Contextual factors

The characterization of barriers and facilitators to intervention implementation are organized by CFIR domain: innovation, inner setting, outer setting, individuals, and process. Table 2 presents representative quotations for each CFIR domain.

**Table 2:** Representative illustrative quotations regarding *APOL1* implementation by Consolidated Framework for Implementation Research (CFIR) domain and construct

CFIR domain	Quotations by construct
Innovation	*Adaptability*: “I think that over time we will evolve to make this practice fit seamlessly into our living donor evaluation process, which is already a process” [GU-NEPH003]
	*Costs (opportunity)*: “I don’t know that there have been any costs other than the time it takes away from other clinical work or clinical time. So, maybe there is a cost there; there’s a give and take of clinical productivity” [NU-NEPH13] “There may be donors who decide not to pursue donation because of their results and the information provided” [NU-NEPH13]
	*Costs* (*financial*): “We’re not paying for the test currently. And the test is, I think, covered fully” [GU-NEPH004]
	*Evidence base*: “The whole point of this is it should affect their willingness to donate either better or worse. But it should. If it has no impact, then why are we doing it, I guess is my point. It’s all about their willingness, but yeah. They should certainly inform their decision-making about donation. That’s the whole point. I think” [NU-NEPH005]
	*Other innovation characteristics*: “Patients will be in shock, especially the two risk variants will increase anxiety, it definitely will increase their anxiety even though we counsel about it” [NU-NEPH001]
Inner setting	*Mission alignment*: “I think it fits well with that global idea of assessing and treating all of our donors as different individuals based on their ancestral background … So, I think that the program is built nicely to have that awareness globally for all of our patients: recipients and donors ….” [NU-NEPH013] *Mission alignment*: “Of course. I mean, I told you we are very aware that 70% of our transplant list, so people who are waiting for kidney transplant or pancreas transplant, are […] Black or African American. And so we are very aware of that. And so anything that we can do to better meet the needs of them and their potential donors, we want to do” [GU-NEPH003]
	*Relative priority*: “We have many different components to the donor program. Ensuring safety of our living donors has always been our highest priority. And this [*APOL1* program] just helps us to do that” [GU-NEPH01]
	*Relative priority*: “I think [*APOL1* testing and counseling] is absolutely a priority. We serve a sizable population of African American donors, African American recipients, and we know that, historically, people of African American ethnicity have not had the full access to transplant as compared to other ethnicities. So I think it’s very important that as a part of the *APOL1* program, we give the opportunities to all our donors to eliminate the discrepancies in access to transplant and access to donation, help them understand and educate them. So I think it is a very important part of the program and helps us in serving our patients better” [GU-NEPH008]
	*Available resources: time:* “The limiting factor is just the time constraints. So, you know, eventually as we start doing this more and more and maybe even screening more people in, on a logistics standpoint, we need to work and make sure that, you know, we’re getting enough time for all components of the donor evaluation” [NU-NEPH004]
	*Structural characteristics*: “I think it’s, well, the perfect place to implement something like this [*APOL1* Program]. We have the people to help. We have the rooms available. We have the number of patients and also, like, patients at risk. So I think it’s something doable at Northwestern …” [NU-NEPH010]
	*Work infrastructure*: “So, our clinic at Georgetown is highly efficient at working up donors … so we already have systems in place to do these things” [GU-NEPH03]
	*Relational connections:* “The need for a dedicated team, the research team, the heavy involvement of the physicians is absolutely necessary” [GU-NEPH08]
	*Relational connections*: “Certainly, cooperation of the different team members … making sure that the nurses, the nephrologist, the assistants are getting those reports. And so, just cooperation between all the team members, I think that’s going to be key” [NU-NEPH003]
Outer setting	*Local attitudes*: “We have a population that is ready to be tested, and they want to be tested” [NU-NEPH010]
	*Local attitudes*: “There’s always been a concern about the health care delivery aspects to the African American community and a lot of the inertial hesitation that rightly comes through because of those issues in the past. So I think the biggest challenge is in delivering; it’s really convincing the patients to move ahead with the testing that I see is the biggest challenge” [GU-NEPH008] *Local attitudes*: “Most of the donors are very open to get the test done. They always want to know their risk factor because, you know, I always trust that it’s based on current data. We cannot predict their future risk factor, like risk of developing CKD. I think one more data will help them, so I think they’re very open to do genetic testing” [NU-NEPH01]
	*Local conditions*: “I think that because we serve such a big population and have a very robust living donor program, we do have several more potential living donors who may be candidates than maybe compared to other programs [that] are smaller in numbers” [GU-NEPH008]
	*Structural/systemic oppression*: “I’m concerned that we’re losing a lot of individuals who don’t want to participate in research because they’re skeptical or worried or not trusting. Those are the people we need in the study” [GU-NEPH003]
	*External pressure*: “I would be incorporating this testing because I want to help people make better decisions about their future health, and I want to feel better about the decisions that we make as a committee when we’re determining if someone could or could not donate at our center if they wanted to. But I don’t view this as an advantage” [GUNEP003]
	*External pressure*: “I do think that provides more holistic care for our donors, and that’s always something we want to do. But I don’t think it’ll provide a competitive advantage for anybody. In fact, it may rule out more donors and put us at a competitive disadvantage, if you think about it that way” [NU-NEPH005]
Individuals	*Leader motivation*: “Our organization supports what we need to make good decisions as a team and to support our donors” [GU-NEPH03]
	*Nephrologist opportunity:* “Sometimes when following up with living donors to share *APOL1* test results with them, they can be difficult to reach. Especially younger people who do not answer their phone” [NU-NEPH001]
	*Donor motivation*: “I think probably some people do not want to get tested, probably because of fear of being positive and then they cannot donate” [NU-NEPH10]
	*Nephrologist capability*: “I think that none of us get trained in genetics how to deliver results and how to support the patients when they have questions related to those results as far as like what do I do with my family, what […] I do with myself. So those will be challenges that I perceive personally” [GU-NEPH04]
Process	“[The intervention] should be protocolized, not so opinion based. So the patient meets these criteria, they should be advised to get tested, this should be the reason to go to a genetic counselor, et cetera, et cetera. So it should be very protocolized so that you give everyone a fair chance and it’s not dependent on the provider” [GU-NEPH02]
	“I think during the clinic, the fact that when I see a patient, they’ve already been introduced to the concept of a *APOL1* versus hearing about it from me for the first time, because they’ve interacted with the Gia. And so they have some idea what I’m going to talk about. And that’s helped” [NU-NEPH003]
	“We definitely needed to educate ourselves better. I don’t think that I could have done it right off without having the [Nephrologist *APOL1* Counseling Training Program] educational modules and the guidance before. So I think that we are just not getting […] any training on genetic diseases or how to navigate this area. So that part I think was necessary to do the educational part, the training part, to be able to do it” [GU-NEPH004].
Sustainability	“I think just because we would like to continue the *APOL1* testing even after, so figuring out reimbursement or insurance coverage for the *APOL1* genetic testing once it’s become protocol and part of our routine workflow. But the test is no longer going to be covered by the study” [NU-NEPH003]
	“I think it would be nice to have access to updated educational materials. Oftentimes, and I’ve seen this recently, I’ve gone back and found educational materials that we have done over the years, and after 3 or 4 years, they’re completely out of date. And so I think that if we had access to up-to-date brochures—not that this brochure would be what we’re going to be handing the patients in 10 years—I think that would be very helpful” [GU-NEPH003]
Equity/ethics	“Many programs would be a little bit hesitant about dissuading donors because you’re going to say that now you’re going to take away donors from African Americans who already have the least living donors. So it’s, there’s two hands to that. Yeah. So it might be the advantage. It depends how you look at it, right? If you want to get more transplants, then the advantage would be not to talk about it” [GU-NEPH002] [*APOL1]* is a high priority because of the population that we serve. People [African American ancestry] are affected in a big way, and they have a lot of other disparities. So, we already have a lot of people with kidney disease. Their families and friends are in their same ancestry usually. So, then the donors are going to be sharing probably some of the similar genetics and ancestry. So, it’s really important that we increase access to donors in a safe way. And I think in the past we were not … that was a big gap, something that we were missing that now we need to fill. So it’s a very, very high priority to make sure that we safely evaluate these donors and offer them everything that we know that’s evidence based for them to make an informed decision [GU-NEPH004]

#### Innovation

Prominent facilitators included low financial *costs *of the intervention, low *complexity*, and *adaptability*. Participants consistently reported that the perceived *cost *of the intervention was a facilitator because the research study covered the cost of the genetic test. Participants described the intervention as “not complicated” overall. Participants considered the potential for the intervention strategy of training nephrologists to engage in culturally competent genetic counseling about *APOL1* to add *complexity* to pre- and post-test LD clinic visits, because they were now expected to provide more information about *APOL1 *to LDs. However, participants acknowledged that providing patient counseling and education was within their overall scope of practice. Participants appreciated the intervention’s *adaptability* in terms of performing the counseling in person or via virtual visits. Participants also expressed appreciation for the intervention’s potential for improving LDs’ understanding of their future risk of CKD post-donation. Participants believed the intervention would achieve the study aims of reducing decisional conflict and improving the informed consent process for LDs, regardless of LDs’ donation decisions.

An innovation barrier was opportunity *costs* because time spent discussing *APOL1* testing meant time lost in clinic that could have been spent on other tasks, as well as the potential for decreasing the number of LDs of African ancestry. Participants reported how the lack of *evidence-based* data about the clinical indication of *APOL1* test results to guide post-test counseling posed a challenge to counseling LDs.

#### Inner setting

Inner setting facilitators included *compatibility, work infrastructure, *and *culture* (particularly the subconstruct *patient-centeredness*). Participants across both sites agreed that the *APOL1 *Program was compatible with their clinic and was easily integrated into the existing clinic workflow. Additionally, the *APOL1* Program aligned with the transplant center’s value of being *patient-centered* by prioritizing their patients’ safety. Participants across both sites reported that *structural characteristics* (e.g., the clinic’s physical layout, IT capabilities) and *work infrastructure* facilitated implementation of the *APOL1* Program. Others appreciated how the Gia chatbot provided additional education to LDs, but the additional time needed for LDs to complete Gia should be factored into the existing clinic workflow.

The most prominent inner setting barriers were *available resources* (particularly the subconstructs of *time*) and *relative priority*. Across both sites, respondents stated that implementing the *APOL1 *Program added time to an already busy clinic. Yet they recognized that time constraints are not insurmountable and can be addressed by adjusting the workflow and appropriately allocating time. Some NU participants perceived the *APOL1* Program as a lower relative priority to other initiatives due to the low volume of LDs of African ancestry. Conversely, GU participants considered the *APOL1* Program as a high relative priority and a facilitator to intervention implementation because of their large volume of LDs of African ancestry.

#### Outer setting

Outer setting facilitators relevant to the *APOL1* Program included *local attitudes* and *market pressure*. Participants reported that favorable *local attitudes* among their patient population facilitated implementation. For example, participants across both sites reported that their LDs were knowledgeable and informed about their risks after using the Gia chatbot. Participants commonly regarded the *APOL1* Program as providing their center an advantage compared to other transplant centers in their region. Some recognized that while the *APOL1 *Program might decrease the number of LDKTs performed, the *APOL1* Program improved patient care by respecting LD autonomy. The most prominent outer setting barrier was *local conditions*, which was related to low LD volume as described above.

#### Individuals

The main individual-level facilitator was high *motivation* to deliver the *APOL1* Program. Participants across both sites reported extensive buy-in from organizational leadership to support the *APOL1* Program.

The most prominent individual-level barrier was nephrologist *opportunity*, defined as the availability, scope, and power to fulfill duties for intervention delivery. After LDs have completed their clinical exams and tests, nephrologists discuss test results with LDs by phone to review their candidacy for donation. As part of the *APOL1* Program, nephrologists are expected to discuss *APOL1* test results and engage in shared decision-making about donation. However, some LDs do not follow up with this clinical discussion, likely because they withdrew from donor evaluation. Participants reported that LDs who do not follow up present a challenge to delivering post-test counseling about *APOL1 *test results. Participants’ lack of confidence in delivering high-quality post-test counseling posed another challenge.

Participants related varied LD perceptions of *APOL1 *testing, with some indicating favorable LD perceptions, though other participants experienced challenges in encouraging donors to undergo *APOL1 *testing. One nephrologist attributed LDs’ hesitancy to fear of not being able to donate if they had two *APOL1* risk variants.

#### Process

Participants across both sites regarded the *APOL1* Counseling Training Program’s educational modules as of high quality and that without them, implementation would not have been possible. The capacity for the Gia chatbot to provide LDs information before their clinic visit and thereby save nephrologists’ time was commonly considered a facilitator.

A key process barrier pertained to the potential negative impact of implementing the *APOL1* Program into an already complex workflow. Participants stated in early interviews that research staff’s screening of LDs for study eligibility upon their arrival to the clinic would sometimes take a few minutes before the LDs met with the nephrologist. Research staff at both sites addressed this concern by screening potential study participants by phone ahead of time. In addition, participants conveyed that the time required for *APOL1* genetic test results to be returned along with LDs’ lack of response to attempts to schedule follow-up appointments to discuss test results remained challenges throughout the study period.

Participants discussed the importance of using intentional communication strategies, such as cross-training nurses and clinic support staff to fulfill multiple roles in the screening and counseling process, and convening regular meetings to evaluate and ensure that the intervention was being delivered equitably and per protocol. Moreover, multiple participants indicated the importance of standardized implementation of the *APOL1 *Program protocol to ensure equitable implementation and reduced bias by eliminating variability in clinical decision-making.

### Sustainability

The most prominent factor affecting sustainability was determining how to cover the costs to support the *APOL1* Program intervention following study completion (e.g., costs of genetic testing as well as staffing to conduct screening; administer Gia; and administer the *APOL1* Counseling Training Program, including continuing medical education and maintenance of certification credits, to new nephrologist faculty). Additionally, participants related that transplant programs would need to invest time and funds to support ongoing training for the faculty and staff, acknowledging that training materials should be updated routinely. Participants anticipated that clinic staff would need additional training to assume administrative tasks that were performed by research staff.

Participants suggested that the costs to sustain the *APOL1* Program would be minimal overall, and they speculated that the genetic test would likely be covered by U.S. Centers for Medicare and Medicaid Services. Others acknowledged that sustaining the *APOL1* Program may be cost-prohibitive because insurance companies do not yet consistently cover *APOL1* genetic testing; thus, additional financial strategies may be needed.

### Changes in perceptions over time

Participants’ perceptions shifted over the course of the study. Initial optimism about the *APOL1* Program’s *potential* to meet study goals transitioned to a *strong conviction* about the *APOL1* Program’s importance for integration into clinical practice. Early apprehension about donor willingness to participate in the study and undergo *APOL1* genetic testing shifted to recognition of effective counseling as a means of improving informed consent within the donor evaluation process. At initial interviews, participants discussed the need for transplant nephrologists to understand the resources required for successful *APOL1* Program implementation. Over time, participants focused on how to continuously improve and sustain the *APOL1* Program from a practical and financial standpoint. Participants explained how the *APOL1 *Counseling Training Program helped improve their counseling, which in turn improved the LD informed consent process. Participants reported not observing a decrease in donation rates, which one participant attributed to improved counseling quality.

### Quantitative results

Across all time points and participants, acceptability, appropriateness, and feasibility ratings were high (Table 3). Among GU and NU participants, mean scores did not change significantly over time. The mean CSAT score for NU participants was 4.97 (range: 3.83–5.53) and was 5.81 for GU participants (range: 5.56–6.56). Combined across both sites, engaged partners was the highest rated subscale, while outcomes and effectiveness was rated as the lowest subscale (Table 4). Participants from GU reported higher CSAT scores overall and across all subscales. Of all participants, eight completed the CSAT, and three had left the institution before CSAT administration. Seven of the eight participants completing the CSAT expressed desire to continue delivering the *APOL1 *Program following study completion. The eighth participant preferred her “own practice” of counseling LDs, recognizing she had “not been doing it [delivering the *APOL1* Program] well” and needed the refresher training she completed.

**Table 3:** Ratings of *APOL1* Program perceived acceptability, appropriateness, and feasibility

**NU nephrologists** (*n* = 5)	**Year 0** *Mean (SD)*	**Year 1** *Mean (SD)*	**Year 2** *Mean (SD)*
AIM	4.85 (0.22)	4.55 (1.01)	4.30 (1.10)
IAM	4.60 (0.42)	4.40 (0.80)	4.25 (0.83)
FIM	4.95 (0.11)	4.55 (1.01)	4.20 (0.51)
**GU nephrologists** (*n* = 3)	**Year 0** *Mean (SD)*	**Year 1** *Mean (SD)*	**Year 2** *Mean (SD)*
AIM	4.31 (0.47)	–	4.33 (0.29)
IAM	4.44 (0.42)	–	4.58 (0.52)
FIM	4.69 (0.47)	–	4.58 (0.52)
**Total (*N*** **=** **8)**	**Year 0** *Mean (SD)*	**Year 1** *Mean (SD)*	**Year 2** *Mean (SD)*
AIM	4.56 (0.44)	–	4.31 (0.84)
IAM	4.47 (0.39)	–	4.38 (0.70)
FIM	4.81 (0.35)	–	4.34 (0.52)

Each measure included four items and was rated on a 5-point ordinal scale

*NU* Northwestern University, *AIM* acceptability of intervention measure, *IAM* intervention appropriateness measure, *FIM* feasibility of intervention measure, *GU* Georgetown University

**Table 4:** Ratings of *APOL1* Program perceived sustainability by Northwestern University (NU) and Georgetown University (GU) participants

CSAT scores Subscale	Total *(N* *=* *8)* (mean)	NU (*n* *=* *5)* (mean)	GU (*n* *=* *3)* (mean)
*Overall CSAT-Short average score:*	5.29	4.97	5.81
Engaged partners	5.91	5.53	6.56
Engaged staff & leadership	5.50	5.40	5.67
Organizational readiness	5.58	5.47	5.78
Workflow integration	5.38	5.20	5.67
Implementation & training	5.29	5.08	5.56
Monitoring & evaluation	4.71	4.13	5.67
Outcomes & effectiveness	4.81	3.83	5.81

The Clinical Sustainability Assessment Tool short form (CSAT-Short) full scale includes 21 items. Each subscale included three items rated on a 7-point ordinal scale

## Discussion

This study evaluated the implementation of the *APOL1 *Program at two large transplant centers, focusing on contextual factors influencing its adoption, implementation, and sustainment. Both sites rated the *APOL1* Program as highly acceptable, appropriate, and feasible over the course of the study. Our findings provide novel insights into the barriers and facilitators encountered during the implementation process, which can inform future use of *APOL1* genetic testing in LD clinical evaluation and of genetic testing in transplantation more broadly.

Prominent facilitators included the *APOL1* Program compatibility with existing clinical processes, the support from organizational leadership, and the perceived benefits of the program in improving LDs’ informed consent and reducing decisional conflict. Another relevant facilitator was the perception of how having either a large or small Black LD volume would increase or decrease, respectively, the relative priority that participating nephrologists placed on the *APOL1* Program. Other research conversely found that a large transplant patient volume hindered the integration of pharmacogenetic testing because of the financial cost of the test and the additional time required to interpret and apply results for many patients [20]. Moreover, the *APOL1 *Program aligned with broader transplant program values of promoting equity and access to transplantation.

The use of the culturally adapted Gia chatbot was highly regarded as an important facilitator for delivering foundational information about *APOL1* and relieving nephrologists to engage LDs in shared decision-making about donation. Our findings align with research evaluating the integration of other genetic tests into clinical practice. Studies evaluating the use of genetic testing among women with breast cancer found that educational resources for patients facilitated implementation and integration of the tests [18, 41]. A large systematic review identified similar facilitators (i.e., high-quality patient and provider educational materials and leadership support of the intervention) influencing the implementation of genomic testing programs and highlighted culturally appropriate communication and available educational resources for patients and providers as important facilitators [21]. Participants’ positive responses to the *APOL1* Program’s educational materials align with research emphasizing professional development to support nephrologist education on counseling patients about genetic testing [22–24].

Prominent barriers included concerns about the lack of clear evidence-based guidelines on the clinical implications of *APOL1* genetic test results to guide donor evaluation. Studies document comparable barriers to the implementation of genetic testing in other clinical contexts [42, 43]. The APOLLO study results will address this concern [12]. Additionally, participating nephrologists expressed concern over the potential for the *APOL1* Program to decrease the number of LDs, as reported elsewhere [13]. Some participants at both sites considered the *APOL1* Program to be a lower relative priority compared to other needs at their transplant program.

Regarding *APOL1* Program sustainability, concerns were expressed regarding clinical time pressures among health care providers. After the study ends, financial support for genetic testing costs and maintenance of access to the Gia chatbot would be needed, as corroborated by other research [21]. Additionally, clinical staff members may need to assume a greater workload to complete tasks covered by the research team. Sustainability planning will invariably focus on this barrier. Activity shifting and staff realignment are successful strategies for addressing this concern through additional training and retraining and by maintaining evaluation and feedback systems [21, 44, 45].

A key strength of this study is its multisite design, which enhances transferability. The longitudinal approach allowed for data collection at multiple time points, providing a comprehensive view of factors affecting implementation over time. The use of CFIR provided a robust theoretical framework for analysis and interpretation. Our mixed-methods approach involving longitudinal qualitative data in combination with longitudinal quantitative data is valuable for providing insights into the magnitude of concerns.

A study limitation was that the sample size of sites and participating nephrologists was relatively small; however, thematic saturation was achieved, which supports credibility. Because participating nephrologists were colleagues of the study investigators, their responses may have been affected by social desirability bias.

Given the growing number of African immigrants to Germany, German transplant centers may need to consider providing tailored quality care to those becoming LDs [46, 47]. This intervention may not entirely translate to Germany, where only close relatives and spouses may legally donate and where only medical specialists certified in genetic counseling may explain results from genetic tests [48]. However, pretest counseling by the transplant team might be feasible.

The study findings have important implications for LDKT and the field of genetic testing in nephrology. Our findings support the feasibility of integrating genetic testing into the LD clinical evaluation process. To sustain the *APOL1* Program, transplant programs should leverage facilitators like the Gia chatbot while addressing identified barriers. Hence, the *APOL1* Program will hold promise in achieving its intended objective of informing and preparing LDs about donation in a culturally sensitive manner. Accordingly, we anticipate that *APOL1* Program implementation will aid in eliminating racialized medicine by focusing attention on genetics rather than race [15].

Suggestions for intervention adaptations include allotting additional time for completing the Gia chatbot during the clinical workflow. Transplant nephrologists should understand that Gia is deliberately delivered immediately before LDs’ nephrologist encounter to prime potential LDs for discussion as a necessary part of pretest counseling. Others suggested delivering additional previsit education, which may increase the feasibility of the *APOL1* Program but would disrupt intervention fidelity. To support future *APOL1* Program implementation, we will disseminate study summaries and make available intervention protocols and strategy descriptions to transplant clinicians, transplant professional organizations, and patient advocacy groups. Tailoring implementation strategies to unique local contextual factors is important. Although several implementation strategies *could* work to address barriers, selecting and tailoring strategies to what the organization perceives to be effective and feasible are equally important.

## Conclusion

Our implementation evaluation of the *APOL1 *Genetic Testing and Counseling Program identified barriers and facilitators. Efforts aimed at addressing the identified barriers and leveraging facilitators by developing intervention- and context-specific implementation strategies may increase the potential for widespread implementation and long-term sustainment. Future research should examine implementation strategies that leverage identified facilitators and mitigate barriers to scale adoption.

## Supplementary Information

CFIR Interview Guide

## Declarations

This study was approved by the NU Institutional Review Board of record (STU00214038) and the GU Institutional Review Board (STUDY00003752) in accordance with the Declaration of Helsinki of 1975*. *The *APOL1* Program was implemented at NU on August 18, 2022, and at GU on August 19, 2022. Eligible participants included all transplant nephrologists, recruited through purposive sampling.
