# Supplementary material for: Contextual factors of implementing APOL1 genetic testing into living kidney donor clinical evaluation
Source: Bundesgesundheitsblatt Gesundheitsforschung Gesundheitsschutz. 2025 Jun 11;68(7):758–68. doi: 10.1007/s00103-025-04068-8 (PMC12254160; doi:10.1007/s00103-025-04068-8)
Supplement: Supplementary file 1 — CFIR Interview Guide [file 103_2025_4068_MOESM1_ESM.pdf]

Supplemental File
CFIR Interview Guide

CFIR Domain	CFIR Construct	Interview Questions	Definition
**Intervention Characteristics**			
	Adaptability	• What kinds of changes do you think you will need to make to the ***APOL1* program** so it will work well in your clinic?<br>• Do you expect the ***APOL1* program** to be adaptable/modifiable in these ways?	The degree to which an intervention can be adapted, tailored, refined, or reinvented to meet local needs.
	Complexity	• How complicated is the ***APOL1* program** to deliver?<br>• What challenges do you anticipate with deploying it?<br>• What challenges do you anticipate with integrating the program into your LD evaluation program?	Perceived difficulty of the intervention, reflected by duration, scope, radicalness, disruptiveness, centrality, and intricacy and number of steps required to implement.
	Evidence Strength & Quality	• What kind of information or evidence are you aware of that shows whether or not the ***APOL1* Program** will work in your setting?<br>• Based on what you know about the ***APOL1* program** do you expect it will achieve the desired outcomes for LDs?	Stakeholders' perceptions of the quality and validity of evidence supporting the belief that the intervention will have desired outcomes.
**Inner Setting**			
	Structural characteristics	• How will the infrastructure of your organization (social architecture, age, maturity, size, or physical layout) affect the implementation of the ***APOL1* Program**?<br>• What kinds of infrastructure changes will need to be made to accommodate the ***APOL1* Program**?	The social architecture, age, maturity, and size of an organization.
	<u>Organizational Climate:</u><br><br>Tension for Change	I'd like you to reflect on the how you and other nephrologists are currently counseling LDs about *APOL1* at your program.<br>• How great is the need to change the way nephrologists counsel LDs about *APOL1*?	The degree to which stakeholders perceive the current situation as intolerable or needing change.
	Compatibility	• To what extent does the ***APOL1* Program** fit with your transplant program's norms, workflows, systems? How does it not fit?	The degree of tangible fit between meaning and values attached to the intervention by involved individuals,

			how those align with individuals' own norms, values, and perceived risks and needs, and how the intervention fits with existing workflows and systems.
	Relative Priority	• To what extent is the ***APOL1* program** a priority for your transplant program compared to other priorities?<br><br>• What makes the ***APOL1* program** (more/less) important than the other priorities?	Individuals' shared perception of the importance of the implementation within the organization.
	Available Resources	• Is the ***APOL1* program** going to require more resources (like time, physical space, and training) than your transplant program currently has available?<br><br>• What resources will the ***APOL1* program** require from your transplant program?<br><br>• How well will your transplant program be able to accommodate those resource needs?	The level of resources dedicated for implementation and on-going operations, including money, training, education, physical space, and time.
**Outer Setting**			
	Market Forces [Peer Pressure]	• To what extent will the ***APOL1* program** give your transplant program an advantage compared to other transplant programs in your area?	Mimetic or competitive pressure to implement an intervention; typically because most or other key peer or competing organizations have already implemented or are in a bid for a competitive edge.
**Process**			
	Doing [Executing]	• What factors will affect your transplant program's ability to implement the ***APOL1* program**?<br><br>• Will your transplant program implement the ***APOL1* program** according to the implementation plan that Northwestern provided, or in some other way?	Carrying out or accomplishing the implementation according to plan. Implement in small phases, steps or cycles of change that cumulatively build, before scaling up more broadly with continued optimization until [Innovation] becomes routine.

	Reflecting and evaluating	• Does your transplant program routinely collect and review data on LDs? What kinds of data?<br>• What kind of information does your transplant program plan to collect as you implement the ***APOL1* program**?<br>• Are you planning on reviewing data collected by the ***APOL1* program** and study in general? What specific metrics do you plan to look at?	Quantitative and qualitative feedback about the progress and quality of implementation accompanied with regular personal and team debriefing about progress and experience. Implementation progress & Innovation progress
**Sustainability** – Subsequent CFIR Interviews ascertain sustainability and include the following questions:			
	Costs	• What costs (including financial and opportunity costs) has the ***APOL1* program** had on your transplant program so far?<br>• What costs (including financial and opportunity costs) do you anticipate the ***APOL1* program** having on your transplant program <u>during the rest of this</u> research study?<br>• What costs (including financial and opportunity costs) do you anticipate the ***APOL1* program** having on your transplant program <u>after</u> this research study ends?	Costs of the intervention and costs associated with implementing the intervention including investment, supply, and opportunity costs.
	<u>Implementation Readiness</u><br><br>Leadership Engagement	• Is there is buy-in from organizational leadership to support the ***APOL1* program** <u>during</u> this research study?<br>• What kind of support have you needed to keep the ***APOL1* program** running <u>during</u> this research study?<br>• What buy-in from organizational leadership do you anticipate needing to support the ***APOL1* program** <u>after</u> this research study ends? What kind of support have you needed for this study?<br>• What kind of support do you anticipate needing to keep this ***APOL1* program** running <u>after</u> this research study ends?	Commitment, involvement, and accountability of leaders and managers with the implementation.
